# Genetic variation for induced and basal resistance against leaf pathogen *Pseudomonas syringae* pv. *tomato* DC3000 among *Arabidopsis thaliana* accessions

**DOI:** 10.1186/s40064-015-1070-z

**Published:** 2015-06-26

**Authors:** Md Motaher Hossain, Farjana Sultana

**Affiliations:** Department of Plant Pathology, Bangabandhu Sheikh Mujibur Rahman Agricultural University, Gazipur, 1706 Bangladesh; College of Agricultural Sciences, International University of Business Agriculture and Technology, Dhaka, 1230 Bangladesh

**Keywords:** Plant growth promoting fungus, Arabidopsis ecotypes, Natural variation, Basal resistance, Induced resistance

## Abstract

In *Arabidopsis thaliana*, significant efforts to determine the effect of naturally occurring variation between phenotypically divergent accessions on different biotic or abiotic stresses are underway. Although it is usually assumed that induced systemic resistance (ISR) against pathogen will covary with plant genetic variation, this assumption has not been tested rigorously in previous experiments. Here, we investigated heritable variation in resistance as well as *Penicillium simplicissimum* GP17-2-mediated ISR to the bacteria *Pseudomonas syringae* pv. *tomato* DC3000 (*Pst*) among a worldwide collection of accessions of *A*. *thaliana*. In this study, 75 Arabidopsis accessions were screened against the bacteria *Pst* following induction and non-induction treatment and their resistance levels were determined by measuring three components of *A*. *thaliana* resistance (infected leaf number, disease severity and pathogen growth). We observed extensive quantitative variation in the number of infected leaves, severity of disease symptoms and the bacterial population size among 75 accessions of *A*. *thaliana* infected with *Pst*. On the contrary, about a two-third of the accessions (49 accessions) showed a reduction in infected leaf number, disease severity and pathogen proliferation after treatment with GP17-2, indicating that GP17-2 induction of resistance is ecotype specific in Arabidopsis. The level of suppression was more pronounced for percent disease severity and pathogen proliferation than for number of infected leaves in ISR-inducible accessions. Accessions non-responsive to GP17-2 treatment generally appeared to be associated with higher basal resistance to infection by *Pst*. Future study with these parental lines employing a variety of crossing schemes may facilitate identification of major trait loci responsible for GP17-2-mediated ISR in Arabidopsis.

## Background

Numerous studies have presented the phenomenon of induced resistance as an effective means to reduce damage of crops by various pathogens. Localized infection that results in protection in spatially separated parts of plants from an unrelated pathogen is called induced resistance or acquired resistance (Ross 1961a, b; Kuć 1987). Induced resistance could be local and systemic. Systemic induced resistance against pathogen lacks the specificity of the hormonal immune system, can be generated by a wide variety of structurally unrelated elicitors, and once activated; it is effective against a wide variety of organisms (Kuć 2006; Hossain et al. 2008). A multitude of potential responses occurs in plants following induction of systemic resistance. Complex sets of signals modulate a suite of responses within a plant to prevent an existing infection from spreading further or to combat secondary infections from a broad spectrum of pathogens. This multicomponent response requires a substantial commitment of cellular resources, including extensive genetic reprogramming and metabolic re-allocation (Somssich and Hahlbrock 1998). Hence, defenses are kept under tight genetic control and are activated only if the plant detects a prospective invader.

Non-pathogenic root colonizing fungi and their microbial metabolites have also shown to induce systemic resistance (ISR) as well against pathogens and insect herbivores. Plant growth promoting fungi (PGPF) are one such group that stimulates plant growth and yield of various crops (Hossain et al. 2008, 2014; Islam et al. 2014a, b). A wide variety of root-associated mutualistic fungi including species belonging to the genera *Trichoderma*, *Fusarium*, *Penicillium*, *Phoma*, *Aspergillus* and sterile fungi are reported to be PGPF (Koike et al. 2001; Hyakumachi and Kubota 2004; Hossain et al. 2008, 2014; Sultana et al. 2008; Horinouchi et al. 2011; Islam et al. 2014a, b). The PGPF isolate *P. simplicissimum* GP17-2 effectively controlled soil-borne diseases (Hyakumachi 1994) and also has been shown to induce systemic defense responses in cucumber plants against several diseases when applied as cell free filtrate (CF) (Shivanna et al. 1996; Koike et al. 2001). The 12,000 Da and lipid fraction in CF of *P*. *simplicissimum* GP17-2 were attributed for stimulating systemic defense responses in cucumber plants (Koike et al. 2001). Using the *Arabidopsis thaliana*-based model system, it has also been demonstrated that treatment with its CF is equally effective as living inocula in inducing ISR against the bacterial leaf pathogen *Pseudomonas syringae* pv. *tomato* DC3000 (Hossain et al. 2007). Arabidopsis is a model plant frequently used in molecular studies on plant–microbe interactions, including systemic acquired resistance and ISR. Some of the characteristics which have facilitated this research, are its small genome size (125 Mb), a rapid life cycle (about 6 weeks), availability of a large number of mutant lines and wild ecotypes. The wild ecotypes, as a result of selection pressures imposed by their different environments of origin, show distinct variation in morphological and physiological traits. In fact, natural variation among genotypes is a prerequisite for biological effects of genetic diversity. The variation in morphological and physiological traits among plant genotypes may also result in variation in relative benefits and efficacy of induced resistance (Walters et al. 2011) and a greater understanding of these dynamic interactions is necessary to facilitate more effective use of elicitors for disease control.

To date, a variety of application methods has been considered to improve the integration of ISR into conventional agriculture, and in some cases with improved efficacy. Several researchers have suggested the need of breeding efforts focused on selection from natural variant ecotypes to add ISR to commercial cultivars. Data exist supporting heritability in the induction of plant defenses and a link between basal resistance and induced resistance (Ton et al. 2001a). This implies that plant genotypes differing in genetically determined basal resistance could differ in the extent to which induced resistance can be expressed. Therefore, it is important to know whether induction of systemic resistance by a particular strain contribute to the suppression of disease in genotypes ranging from susceptible to resistant. Until now only a few studies have examined genotypic effects of domesticated crops or their wild relatives on ISR (Walters et al. 2011). From *A*. *thaliana* model system, a great deal has already been known about the molecular mechanisms of induced resistance, mostly because of mutational screens. Yet, it is unclear what variability exists among the various ecotypes of *A*. *thaliana* for induced resistance responses, except in a limited case involving a single ISR-eliciting PGPR strain *Pseudomonas fluorescens* WCS417r, which is so far the only known such study (Van Wees et al. 1997; Ton et al. 1999). Moreover, ISR consequences of natural variation in plants have not yet been investigated using any PGPF. In the present study, we investigated heritable as well as inducible variation in resistance to the bacteria *Ps*. *syringae* among a wide-ranging collection of accessions of *A*. *thaliana*. In this study, we measured three components of host resistance (infected leaf number, disease severity and pathogen growth) and determined their relative importance to *P*. *simplicissimum* GP17-2-mediated ISR. Finally, analysis was done to find out the correlation between the ISR noninducible phenotype of Arabidopsis accessions and level of basal resistance against *Pst*. Screening of a set of 75 accessions in the current study for induced and basal resistance will be useful to appraise the variation in the interaction phenotypes of various Arabidopsis ecotypes observed upon pretreatment with GP17-2 and inoculation with *Pst*, opening the possibility of breeding plants with improved inducible resistance responses.

## Methods

### Plant materials and growth condition

A total of 75 *A*. *thaliana* accessions was provided by H. Koyama (Gifu University, Gifu, Japan) and used in the present study. The accessions were originally sourced from RIKEN Bioresource Center. The accession names were listed in Table 1, where the JA number is the stock number of the RIKEN Bioresource Center (Table 1). Arabidopsis plants were grown in hydroponic culture. Hydroponic culture was carried out using a culture apparatus, which was developed by Toda et al. (1999). This apparatus contains a plastic photo slide mount (Fuji film, Japan) and a mesh (50 holes per inch) made of thick nylon wire. The basic culture solution consisted of MGRL nutrients at a concentration of 10^−1^ (Fujiwara et al. 1992). Seeds of Arabidopsis were soaked in 0.5 mL of distilled water in Eppendorf tubes and kept in a refrigerator for 3 days at 4°C to synchronize germination. Thirty seeds, ten in each line were then placed on the apparatus. All apparatus were floated on 6 L of nutrient solution and kept in the growth room at 20°C with 14 h daytime and 10 h night cycles. The nutrient solution was renewed every 7 days and the culture was continued for 3 weeks.

**Table 1:** Arabidopsis accessions screened for *P*. *simplicissimum* GP17-2-mediated ISR against *Ps*. *syringae* pv. *tomato* DC3000

Serial number	Accession name	Stock number	Serial number	Accession name	Stock number
1	Aa-0	JA1	39	Dra-2	JA292
2	Ag-0	JA2	40	Edi-0	JA69
3	Ak-1	JA3	41	El-0	JA72
4	An-2	JA265	42	Ei-2	JA70
5	Ba-1	JA8	43	En-2	JA73
6	Bay-0	JA9	44	Ep-0	JA74
7	Bd-0	JA	45	Ge-1	JA90
8	Be-0	JA12	46	Gr-6	JA308
9	Be-1	JA269	47	Hn-0	JA108
10	Blh-0	JA18	48	Is-0	JA111
11	Bla-1	JA14	49	Jl-5	JA314
12	Bla-12	JA16	50	Jm-0	JA115
13	Bla-2	JA270	51	Kas-1	JA119
14	Bla-3	JA271	52	Kl-5	JA124
15	Br-0	JA21	53	La-1	JA131
16	Bs-1	JA22	54	Ler-0	NW20
17	Bs-5	JA23	55	Cvi-0	N8580
18	Bsch-0	JA24	56	Ler-2	N8581
19	Bsch-2	JA277	57	Li-2	JA135
20	Bu-0	JA25	58	Li-2-1	JA136
21	Bu-14	JA33	59	Mh-0	JA150
22	Bu-15	JA35	60	Ms-0	JA157
23	Bu-17	JA36	61	Mt-0	JA158
24	Bur-0	JA44	62	Old-2	JA177
25	Cal-0	JA48	63	Oy-0	JA181
26	Can-0	JA49	64	Pa-1	JA182
27	Cha-0	JA51	65	Pn-0	JA190
28	Chi-0	JA53	66	Po-0	JA349
29	Cit-0	JA55	67	Po-1	JA191
30	Cl-0	JA56	68	Ra-0	JA195
31	Col-0	JA58	69	Rak-2	JA196
32	Col-4	N933	70	Rd-0	JA197
33	Col-gl1	N1644	71	Rsch-4	JA201
34	Da-0	JA61	72	UK-3	JA241
35	Db-0	JA62	73	Van-0	JA243
36	Di-0	JA63	74	Ws-0	JA252
37	Do-0	JA66	75	Xxx-0	JA261
38	Dr-0	JA67			

### The PGPF isolate *P. simplicissimum* GP17-2 and preparation of its CF

The PGPF *P. simplicissimum* GP17-2 was isolated from the rhizosphere of zoysiagrass (*Zoysia tenuifolia* L.). The fungus was grown in PDA media for 7 days. Twenty mycelial disks of GP17-2 culture were obtained from the growing margin of a colony on PDA and transferred to a 500 mL Erlenmeyer flask containing 200 mL potato dextrose broth (PDB). The fungus was cultured without shaking at room temperature (25°C) for 10 days. The crude culture filtrate was separated from mycelia and filtered through two layers of Whatman No. 2 filter. The filtrate was then filter sterilized (0.22 μm Millipore filters, Millipore products division, Bedford, USA).

### Induction treatment of plants with CF from *P*. *simplicissimum* GP17-2

Two-week old seedlings grown in hydroponic culture were treated with CF from *P*. *simplicissimum* GP17-2. Induction treatment of plants was performed 1 day before challenge inoculation by dipping the roots of seedlings in 50% diluted CF of GP17-2 for 1 h. Control plants were treated with 50% diluted PDB. Treated roots were washed thrice with sterilized distilled water and plants were returned to the hydroponic system with the same conditions as described above.

### Pathogen inoculation and disease assessment

The rifampicin resistant virulent bacterium *Ps. syringae* pv. *tomato* DC3000 (*Pst*) provided by Y. Ichinose (Okayama University, Okayama, Japan) was used as pathogen for inoculating Arabidopsis plants. Plants were challenge inoculated 1 day after induction treatment with CF of GP17-2. The virulent bacterial pathogen *Pst* was cultured in liquid King’s medium B (KB) at 28°C. After overnight incubation, cells were collected by centrifugation, washed twice and re-suspended in 10 mM MgSO_4_. The plants were placed at 100% relative humidity 1 day before challenge inoculation. Plants were inoculated by spraying the *Pst* suspension containing, 2.5 × 10^7^ cfu mL^−1^ bacteria and 0.01% (v/v) Silwet L-77 (Nihon Unica, Tokyo, Japan) as a detergent on to the rosette leaves, until run off. The inoculated plants were kept at 100% relative humidity in darkness for 2 days in order for disease to develop. Plants were then transferred to a growth room. Five days after pathogen challenge, disease severity was measured for each plant by recording the percent infected leaves, percent of total plant leaf surface showing symptoms from 0 = no symptoms to 100 = most severe with necrotic symptoms (Ryu et al. 2003; Hossain et al. 2007). The number of *Pst* in inoculated leaves was assessed by collecting all leaves separately from three replications. Leaves were then weighed, rinsed thoroughly in sterile water and homogenized in sterilized distilled water (Pieterse et al. 1998; Hossain et al. 2007). Subsequently, appropriate dilutions were plated onto KB media supplemented with 50 mg L^−1^ rifampicin. After 48 h of incubation at 28°C, the number of rifampicin-resistant colony-forming units (cfu) per gram of infected leaf tissue was determined.

### Statistical analysis

The experimental design was completely randomized, consisting of three replications for each treatment. The experiment was repeated two to three times and treatment means obtained were separated using a Student’s t test. Susceptibility categorical data were expressed as number and percentages of accessions and were analyzed via Chi square (χ^2^) analysis. Correlation coefficients as well as simple linear regression analysis were done by using the Analysis Tool Pak in Microsoft Excel. All analyses were done at *P* < 0.05.

## Results

### Screening of Arabidopsis accessions for GP17-2-mediated ISR with regard to the percent infected leaves per plant

**Figure 1:**
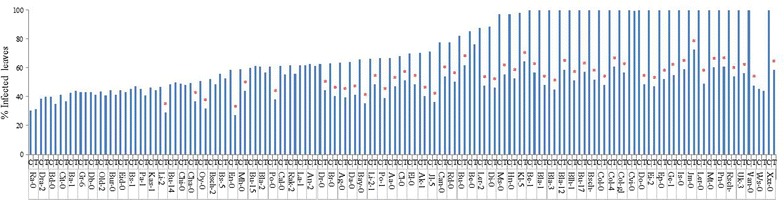
Arabidopsis accessions showing percent infected leaves caused by *Ps*. *syringae* pv. *tomato* DC3000 infection in *P*. *simplicissimum* GP17-2-treated and control plants. ISR was induced by treating the roots of 2-week-old plants with CF of *P*. *simplicissimum* GP17-2. *Pst* was sprayed onto leaves until run-off. Percent infected leaves were measured 5 days after challenge inoculation. In individual accession *asterisk* indicate a statistically significant difference between treatments (Student’s t test, *P* < 0.05). The data presented are from representative experiments that were repeated three times with similar results.

**Figure 2:**
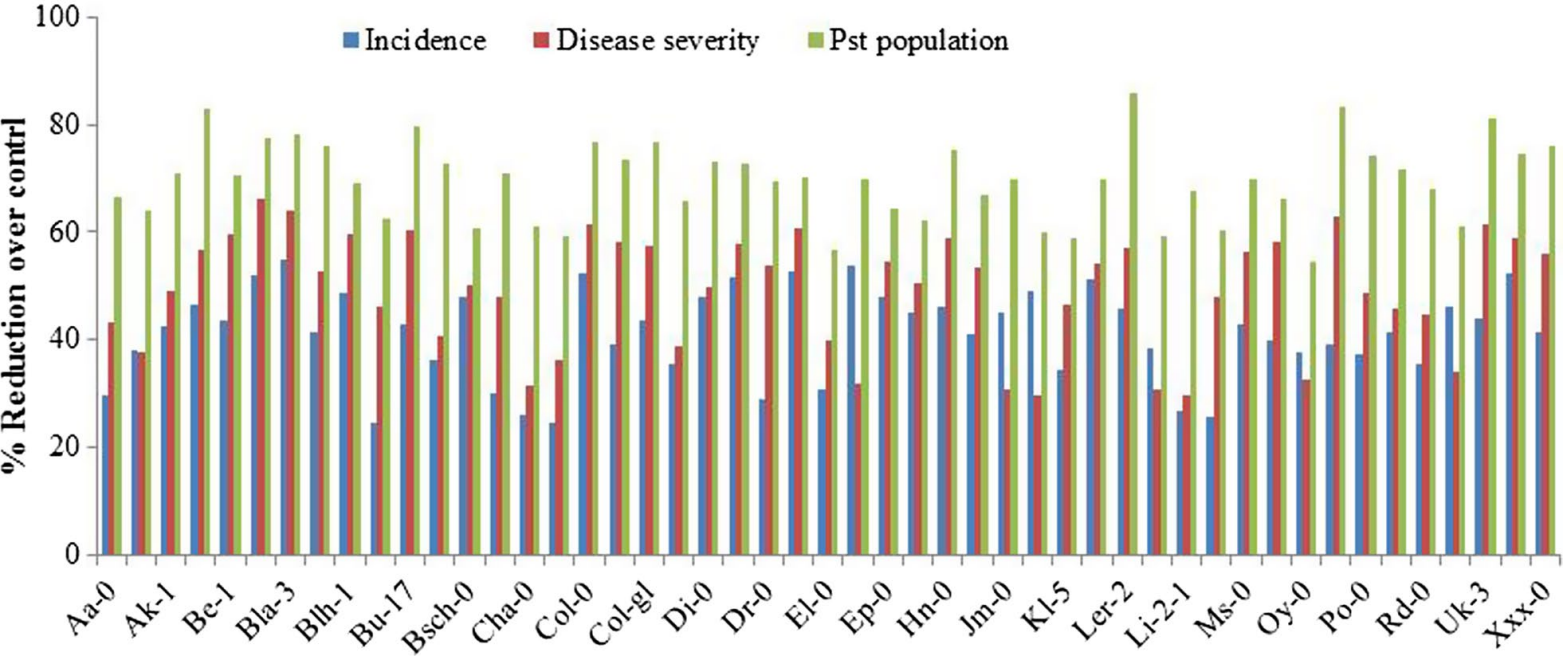
*P*. *simplicissimum* GP17-2-mediated ISR inducible Arabidopsis accessions showing percent reduction in infected leaf numbers, disease severity and pathogen population over control-treated plants after infection by *Ps*. *s*. *tomato* bacterium. The percent reduction in infected leaf numbers and *Pst* population due to GP17-2 treatment over control was calculated as follows: % Reduction = [1 − (Value in treated plant/Value in control plant)] × 100.

Five days after challenge with *Pst* pathogen, the plants developed typical bacterial speck disease symptoms, consisting of necrotic or water-soaked spots surrounded by extensive chlorosis. Induced protection against the pathogen was quantified by determining the percent infected leaves per plant. Our results indicated a wide variation among the Arabidopsis accessions for percent infected leaves both as basal and induced resistance marker. As shown in Figure 1, percent infected leaves in control-treated plants varied over a range of 30–100% in the different accessions, where the lowest percentage of infected leaves was observed in Ra-0 and the highest was found in several accessions including Col-0. Similarly, accessions were differentially responsive to GP17-2 treatment in reducing the number of *Pst*-infected leaves compared to non-treated plants. Among the 75 accessions tested, 49 showed (65%) a significant reduction in percent-infected leaves per plants. The percent-infected leaves in GP17-2-treated plant ranged from 24 to 99%, where accession with the fewest percent infected leaves was En-0 and accession exhibiting greatest number of infected leaves was Cvi-0. In GP17-2-inducible ecotypes, the reduction in percent infected leaves was recorded to be 25–55% and was consistent in three independent experiments. The greatest reduction in percent infected leaves due to GP17-2 treatment was recorded in Bla-3 (55%) and the lowest was in Bu-0 (Figure 2).

### Screening of Arabidopsis accessions for GP17-2-mediated ISR with regard to the percent disease severity per plant

**Figure 3:**
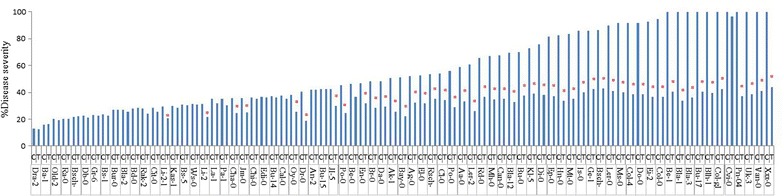
Arabidopsis accessions showing disease severity caused by *Ps*. *syringae* pv. *tomato* DC3000 infection in *P*. *simplicissimum* GP17-2-treated and control plants. ISR was induced by treating the roots of 2-week-old plants with CF of *P*. *simplicissimum* GP17-2. *Pst* was sprayed onto leaves until run-off. Disease severity was measured 5 days after challenge inoculation by recording the percentage of total leaf surface showing symptoms for each plant, where 0 = no symptoms; 100 = most severe necrotic symptoms. In individual accession *asterisk* indicate a statistically significant difference between treatments (Student’s t test, *P* < 0.05). The data presented are from representative experiments that were repeated three times with similar results.

Results in Figure 3 show that percentage area of leaf with symptoms by the disease lesions (disease severity) varied across the non-treated and GP17-2-treated Arabidopsis accessions screened. The disease severity amongst control-treated accessions ranged from 12 to 100%. There was a high severity of the disease on several accessions including Col-0, Be-1, Bla-1, Bla-3, Blh-1, Bu-17, Col-gl, Cvi-0, Pn-0, Uk-3, Van-0 and Xxx-0 accession. The accessions showing the lowest disease severity was Dra-2. The severity of the disease symptoms in many of these accessions were greatly reduced by GP17-2 treatment. Our results indicated that Arabidopsis accessions treated with GP17-2 were generally more resistant to foliar diseases caused by *Pst* and the disease severity in ISR-expressing and non-expressing plants ranged from 12 to 44%. However, a total of 49 (65%) GP17-2-treated accessions exhibited a significant reduction in disease severity compared with non-induced control plants, showing a 29–66% lower proportion of leaf surface with disease symptoms. Among these accessions, Bla-1 expressed the highest reduction in disease severity, while Jl-5 was the accession that exhibited the lowest reduction after GP17-2 treatment (Figure 2).

### Screening of Arabidopsis accessions for GP17-2-mediated ISR with regard to the foliar population of *Pst*

**Figure 4:**
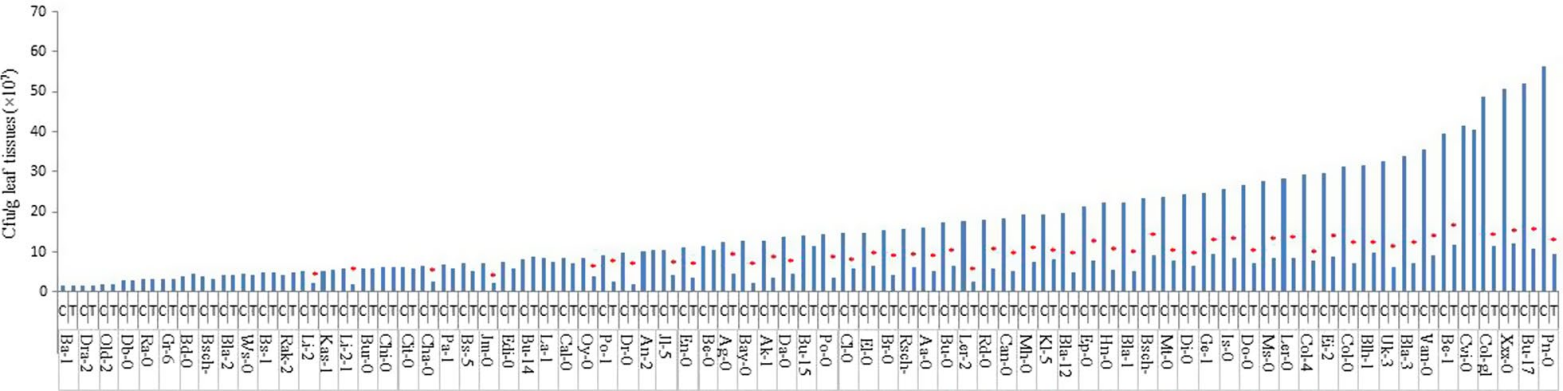
Arabidopsis accessions showing number of *Ps*. *syringae tomato* bacterium in leaves of *P*. *simplicissimum* GP17-2-treated and control plants. Data are presented as numbers of cfu g^−1^ fresh weight, each from three sets of eight whole shoots harvested 5 days after challenge inoculation with virulent pathogen *Pst*. In individual accession *asterisk* indicate a statistically significant difference between treatments (Student’s t test, *P* < 0.05). The data presented are from representative experiments that were repeated twice with similar results.

Determination of the number of colony-forming units (cfu) of *Pst* in challenged leaves revealed that size of foliar population of *Pst* likewise varied among accessions both in control and GP17-2 treated plants. In control-treated accessions, *Pst* population ranged between 1.53 × 10^7^ and 56.43 × 10^7^ cfu/g leaves, where the highest *Pst* population was found in Pn-0, while Ba-1 exhibited the lowest (Figure 4). However, proliferation of *Pst* was significantly inhibited due to GP17-2 treatment in the same 49 accessions that showed significant reduction in infected leaf number and disease severity. Compared with control-treated plants of these accessions, GP17-2-treated plants showed a 53–84% decrease in growth of *Pst* in challenged leaves, demonstrating that level of suppression was more pronounced for pathogen proliferation than for number of infected leaves and percent disease severity (Figure 2). Accession with maximum decrease in growth of *Pst* was Ler-2, while Oy-0 exhibited the lowest reduction. Clear differences in the inhibition of bacterial proliferation in leaves of GP17-2-treated plants indicate that symptom reduction is associated with inhibition of bacterial proliferation (Figure 4).

### Correlation between disease incidence or symptom severity and bacterial population

**Figure 5:**
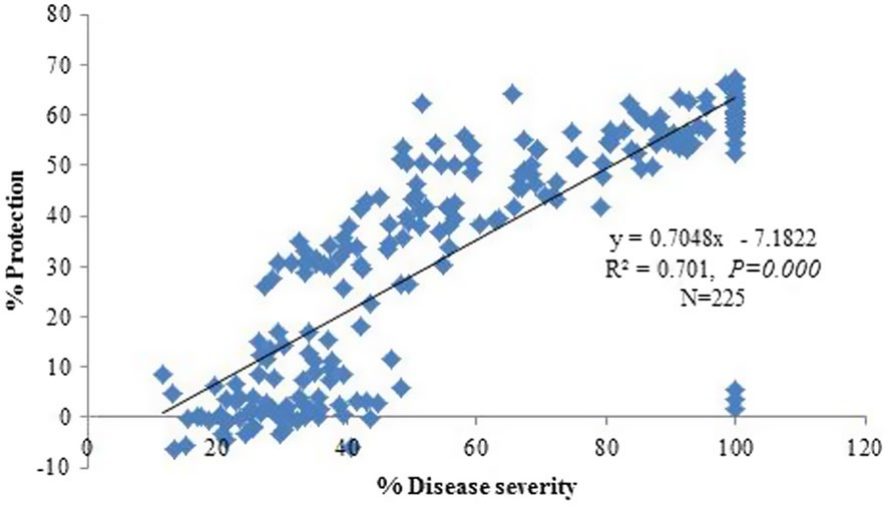
Correlation between the percent disease severity in control-treated accessions and the resulted percent reduction in disease severity in the same plants conferred by *P*. *simplicissimum* GP17-2 treatment. Three data points are shown for each of 75 plant lines in the scatterplot, N = 75 × 3 = 225. The percent reduction in disease severity due to GP17-2 treatment was calculated as follows: % Reduction = [1 − (Disease severity in treated plant/Disease severity in control plant)] × 100.

Analysis was made to determine how far expression of GP17-2-mediated ISR depends on the level of basal resistance in Arabidopsis ecotypes to *Pst*. Percent infected leaves and symptom severity due to *Pst* infection are equivalent phenotypic indicators of host basal resistance to the bacterium. When a large number of accessions is used, the estimation of infected leaf number will considerably save time compared to that of disease severity. However, the variation in basal resistance with respect to disease severity was much wide ranging than to number of infected leaves. Therefore, disease severity provides more appropriate phenotype of basal resistance in ecotypes to *Pst* infection. Based on the mean disease severity in control-treated plants, individual accession was hypothetically categorized as either highly susceptible, moderately susceptible (70–40%) or lowly susceptible (<40%). Infection phenotypes with disease severity >70% were classified as highly susceptible and those with disease severity of 70–40% and <40% were classified as moderately and lowly susceptible, respectively (Table 2). Out of 75 accessions evaluated, 25 accessions were found to fall within highly susceptible group. Of the remaining accessions, 23 accessions were moderately susceptible and 27 were lowly susceptible. Among the 25 highly susceptible accessions, 24 (96%) showed significant level of GP17-2-mediated ISR against *Pst*, when disease severity was compared between GP17-2-treated and non-treated plants (Table 2). Similarly, out of 23 moderately susceptible accessions group, 20 (86.95%) showed significantly reduced disease severity in GP17-2-treated plants compared to non-treated plant (Table 2). On the other hand, only 5 (18.5%) of 27 lowly susceptible accessions were responsive to GP17-2-treatment in showing significant level of ISR against the bacterium (Table 2). These indicate that most plants have higher expression of GP17-2-mediated ISR despite higher degrees of infection. The Chi Square (χ^2^ = 10.36; df = 2; 0.001 < *P*<0.010) test revealed a significant difference in ISR-responsiveness among different *Pst* susceptibility accession groups in Arabidopsis. In other words, ISR-responsiveness was not equally distributed across the different levels of host susceptibility in Arabidopsis accessions towards *Pst*. Accessions that had higher *Pst* susceptibility were likely to be more ISR-responsive than accessions that had less susceptibility. In order to elucidate a clear functional relationship between the host basal resistance and the GP17-2-induced ISR responsiveness of Arabidopsis ecotypes, we calculated the correlation between the symptom severity in control-treated ecotypes and the reduction in symptom severity in GP17-2-treated plants. There was a significant positive relationship observed between the symptom severity in control-treated ecotypes and the reduction in symptom severity due to GP17-2 treatment in the same plants, r (223) = 0.84, *P* < 0.000. That means percent reduction in symptom severity in ISR-inducible ecotypes linearly increased as percent disease severity increased in control-treated plants. Among all accessions, 70% of the values fit the model (Figure 5). These results indicate the ISR-inducible phenotype of Arabidopsis ecotypes was highly correlated with a relatively low level of basal resistance against *Pst*.

**Table 2:** Response of Arabidopsis accessions to *Ps*. *syringae* pv. *tomato* DC3000 infection and *P*. *simplicissimum* GP17-2-mediated ISR

Groups (based on percent disease severity)	Number of accessions	Number of ISR responsive accessions	% Accessions expressing ISR	Chi square test (χ^2^)	df	*P* value^a^
Highly susceptible (DS >70%)	25	24	96.00	10.36	2	0.0056
Moderately susceptible (DS = 40–70%)	23	20	86.95			
Lowly susceptible (DS <40%)	27	5	18.51			

*DS* %disease severity.

^a^ *P* < 0.05 means there is significant difference.

## Discussion

The capacity to express *P*. *simplicissimum* GP17-2 mediated ISR was found to be dependent on the plant genotype. Selection of naturally occurring variants that were higher resistant to disease could be useful for future breeding program. Useful genes for plant breeding are already abundant. In the present study, 75 different Arabidopsis ecotypes were screened to develop an understanding of naturally occurring variation in ISR inducibility and basal resistance against *Pst*. From our results, it clearly appears that the number of *Pst*-infected leaves, the proportion of leaf surface area with the disease symptom and the size of *Pst* population in infected leaves were equivalent phenotypic indicators of both inducible and basal host resistance to the bacterium. Arabidopsis ecotypes were differentially responsive in expressing basal resistance as well as the GP17-2-mediated ISR, when compared these phenotypic indicators during infection by *Pst* bacterium. The ecotypes ranged from highly susceptible to highly resistant to *Pst* infection. Likewise, they were responsive to completely non-responsive to GP17-2 treatment in reducing *Pst* infection, suggesting that specific interactions between the GP17-2 and the plant ecotypes determine ISR in Arabidopsis. For instance, 49 of the tested ecotypes including Col-0 and Ler-0 were responsive to induction of ISR by GP17-2, resulting in a significant suppression of the disease caused by *Pst* in these plants. In ISR-inducible ecotypes, although induced resistance was expressed as a reduction in infected leaf numbers, symptom severity and *Pst* proliferation, the level of reduction varied for each ISR trait. Our results indicate that GP17-2-mediated ISR had more severe effect on *Pst* population than in infected leaves and disease severity. On the contrary, 27 ecotypes including Cvi-0 did not develop ISR after treatment with GP17-2. This observation commemorates a previous conclusion that capacity to trigger ISR is ecotype-dependent in Arabidopsis (Ton et al. 1999, 2001a, b; Van Wees et al. 1997). In that study, ISR elicited by *Ps. fluorescence* WCS417r against *Pst* was clearly associated with ecotype-specific. In carnation, cultivar specificity with regard to expression of WCS417r-mediated ISR has also been reported (Van Peer et al. 1991). Rhizobacteria-mediated ISR against *Pst* in Arabidopsis required the presence of a single dominant gene and the corresponding locus, designated as *ISR1* was mapped between markers B4 and GL1 on chromosome 3 (Ton et al. 1999). Therefore, the observed non-responsiveness in 27 ecotypes to express GP17-2-mediated ISR suggests the absence of (a) genetic determinant(s) essential for induction and expression of ISR.

The level of basal resistance can influence the extent to which GP17-2-mediated ISR is expressed. To investigate how far expression of GP17-2-mediated ISR depends on the level of basal resistance understand the relationship between ISR responsiveness and basal resistance to pathogen, we classified the accessions arbitrarily on the basis of disease severity. In these groupings, majority (95.4%) of highly susceptible accessions were ISR responsive, ruling out the possibility that ISR was masked by the high susceptibility of these ecotypes to *Pst*. On the other hand, highly resistant ecotypes were commonly ISR-noninducible, meaning that expression of GP17-2-mediated ISR is less consistent when basal resistance of these ecotypes to *Pst* is high. These results are in agreement with previously obtained results of Liu et al. (1995) who demonstrated that two susceptible cucumber cultivars expressed ISR after treatment with *Serratia marcescens* 90–166, while a resistant cultivar did not. Likewise, the WCS417r non-induced Ws plants infected with *Phytophthora parasitica* did not exhibit enhanced disease susceptibility compared to ISR-expressing Col-0 (Ton et al. 2001b). Therefore, the observed phenomenon that Arabidopsis ecotypes capable of expressing ISR have a substantially lower level of basal resistance to *Pst* than ecotypes impaired in their ISR response strongly suggests that GP17-2-mediated ISR in Arabidopsis does not utilizes components of the basal resistance pathway. Apparently, both defense mechanisms are genetically distinct resistance responses. However, several situations have been reported in which an opposing association between the capacity to express ISR and the level of basal resistance against the challenging pathogen was existed. For instance, ISR induced by WCS417r against fusarium wilt, caused by *Fusarium oxysporum* f. sp. *dianthi*, was clearly expressed in the moderately resistant cultivar Pallas, but less strongly and consistently in the susceptible cultivar Lena (Van Peer et al. 1991). Similarly, the WCS417r-mediated ISR-noninducible phenotype in Arabidopsis correlated with a relatively low level of basal resistance against *Pst* (Ton et al. 2001b). Moreover, both susceptible and resistant cultivars of radish were capable of expressing *Ps*. *fluorescens* WCS374-mediated ISR against fusarium wilt to the same extent (Leeman et al. 1995). These demonstrate that a clear correlation between the capacity to express ISR and the level of basal resistance against the challenging pathogen was absent in many instances. However, most of the aforementioned studies used only a limited number of cultivars or ecotypes in contrast to the present study which had done a more comprehensive analysis using a large number of ecotypes and delivered a definitive understanding of their naturally occurring variation in ISR inducibility and basal resistance against *Pst*.

In this study, we studied ecotype specificity of GP17-2-mediated ISR in Arabidopsis in relation to the level of ecotype-specific basal resistance against *Pst*. The GP17-2-nonresponsive phenotype correlates with a relatively high level of basal resistance against *Pst*. These studies prepared the ground for future studies to clearly show the mechanistic response of resistance or susceptibility in these accessions against *P*. *syringae*. Further studies in the genetic analysis of variation in the ISR expression ability may contribute to a better understanding of its role in affecting quantitative trait variation at the phenotypic level.

## Conclusion

Plant growth promoting fungus *P*. *simplicissimum* GP17-2 induction of resistance against the leaf pathogen *Ps. s.* pv*. tomato* DC3000 (*Pst*) was ecotype specific in Arabidopsis. The GP17-2- nonresponsive phenotype correlated with a relatively high level of basal resistance against *Pst*. Accessions non-responsive to GP17-2 treatment were generally appeared to be associated with higher basal resistance to infection by *Pst*. This study will help exploit genetic variability within populations of Arabidopsis to improve the utility of ISR in the field.

## Abbreviations

PGPF: plant growth promoting fungi
ISR: induced systemic resistance
*Pst*: *Pseudomonas syringae* pv. *tomato* DC3000
